# A randomized, cross-over trial assessing effects of beverage sodium concentration on plasma sodium concentration and plasma volume during prolonged exercise in the heat

**DOI:** 10.1007/s00421-022-05025-y

**Published:** 2022-09-29

**Authors:** L. A. J. Wijering, J. D. Cotter, N. J. Rehrer

**Affiliations:** School of Physical Education Sport and Exercise Sciences, Otago University, P.O. Box 56, Dunedin, 9054 New Zealand

**Keywords:** Hyponatremia, Electrolytes, Fluid balance, Hydration

## Abstract

**Purpose:**

This study assessed whether increasing sodium in a sports drink above that typical (~ 20 mmol L^−1^) affects plasma sodium and volume responses during prolonged exercise in the heat.

**Methods:**

Endurance trained males (*N* = 11, 36 ± 14 y, 75.36 ± 5.30 kg, ${\dot{\text{V}}}$O_2max_ 60 ± 3 mL min^−1^ kg^−1^) fulfilled requirements of the study including one 1-h exercise pre-trial, to estimate fluid losses (to prescribe fluid intake), and two, experimental trials (3-h or until tolerance), in random order, cycling (55% ${\dot{\text{V}}}$O_2max_, 34 °C, 65% RH). Beverages contained 6% carbohydrate and either 21 mmol L^−1^ (Low Na^+^) or 60 mmol L^−1^ sodium (High Na^+^). Analyses included linear mixed models and *t*-tests.

**Results:**

Cycling time was similar 176 ± 9 min (Low Na^+^); 176 ± 7 min (High Na^+^). Fluid intake was 1.12 ± 0.19 L h^−1^; 1.14 ± 0.21 L h^−1^, resp. Body mass change was − 0.53 ± 0.40%;  − 0.30 ± 0.45%, resp. Sodium intake was 69 ± 12 mmol; 201 ± 40 mmol, resp. Plasma sodium concentration was greater in High Na^+^ than Low Na^+^ (*p* < 0.001); decreasing in Low Na^+^ (− 1.5 ± 2.2 mmol L^−1^), increasing in High Na^+^ (0.8 ± 2.4 mmol L^−1^) (*p* = 0.048, 95% CI [− 4.52, − 0.02], *d* = 0.99). Plasma volume decreased in Low Na^+^ (− 2 ± 2%) but remained unchanged in High Na^+^ (0 ± 3%) (*p* = 0.01, 95% CI [− 3.2, − 0.5], *d* = 0.80).

**Conclusions:**

When conducting prolonged exercise in the heat, those who fully hydrate would benefit by increased sodium content of the beverage by improved plasma volume and sodium maintenance.

Australian New Zealand Clinical Trials Registry (ACTRN12616000239460) 22/02/16.

**Supplementary Information:**

The online version contains supplementary material available at 10.1007/s00421-022-05025-y.

## Introduction

The role of sodium ingestion on the development of exercise associated hyponatremia (EAH) is uncertain (Hew-Butler et al. 2017). Evidence from controlled studies of prolonged exercise in the heat indicates that sodium intake, both acutely (Vrijens and Rehrer 1999) and chronically (Koenders et al. 2017), can significantly affect plasma sodium concentration during exercise. Sodium balance is important with respect not only to risk of hyponatremia but also to retaining water and reinstating hydration after significant sweat losses (Merson et al. 2008; Shirreffs et al. 1996). In most sport drinks, sodium concentration is considerably less (Chatterjee and Abraham 2019), than the typical sweat sodium concentration (Baker 2017). The usual carbohydrate and electrolyte (primarily sodium) content (~ 6% carbohydrate, ~ 20 mmol L^−1^ sodium) of a sports drink made to be consumed during exercise is designed to balance hydration with substrate provision. This composition is based on the efficacy of the carbohydrate, in terms of supplementing limited endogenous reserves (Burke et al. 2011), concentration effects on gastric emptying, osmolarity effects on net fluid absorption (Rehrer et al. 1992) and, possibly most importantly, palatability (Coombes and Hamilton 2000). Thus, consuming sports drinks to replace sweat losses that are substantial normally results in a net loss of sodium, which can become functionally significant. In a previous study in our lab, during prolonged (~ 3 h) exercise (55% ${\dot{\text{V}}}$O_2max_) in the heat (34 °C), plasma sodium concentration decreased with a sports drink (18 mmol L^−1^ sodium), although to a lesser extent than with water, when fluid losses (~ 1.4 L h^−1^) and intakes were high (~ 1.2 L h^−1^) and net body mass loss low (~ 0.8%) (Vrijens and Rehrer 1999). In another study with mixed, low to moderate intensity exercise (3 h mixed walking and cycling, 130–140 bpm + 8 calf raises + 45 min walking 5.5 km/h at a 12% grade), in slightly milder conditions (30 °C), with similar fluid ingestion, a beverage containing 20 mmol L^−1^ sodium was compared with one containing 36 mmol L^−1^ (Anastasiou et al. 2009). Both were superior to beverages without sodium, but were not different from each other. Replacing fluid losses without replacing sodium losses has been shown to limit the extent of rehydration and plasma volume recovery (Shirreffs et al. 1996; Maughan and Leiper 1995). A reduction, magnitude dependent, can decrease cardiac output and/or increase heart rate and, thereby, physiological and perceived strain during exercise, as well as impair peripheral blood flow and thermoregulation (Nadel et al. 1980; Strydom and Holdsworth 1968). In contrast, when plasma volume is enhanced the strain is mitigated (Sims et al. 2007). Providing sodium in a beverage during exercise has also been shown to limit plasma volume decrease during exercise, compared to when no sodium was ingested, but no difference was observed between providing 20 mmol L^−1^ with 36 mmol L^−1^ (Anastasiou et al. 2009).

Therefore, the purpose of the present study was to evaluate plasma sodium and plasma volume responses to prolonged exercise in the heat with fluid losses compensated by a typical commercially available sports drink (21 mmol L^−1^ sodium) versus the same beverage but with increased sodium (60 mmol L^−1^). It was hypothesized that the 60 mmol L^−1^ beverage would maintain plasma sodium, and thereby also volume, to a greater extent than the 21 mmol L^−1^ beverage.

## Methods

A double blind, randomized cross-over design was employed. Randomization was achieved via an on-line, random number generator. A technician allocated the treatment and assigned a letter to each. Researchers did not know which letter was which treatment. This study was approved by the University of Otago Human Health Ethics Committee (H16/002).

### Pre-trials

Participants gave written informed consent and filled in an activity and health questionnaire (PAR-Q). Body mass and height were measured and, thereafter, a graded maximal oxygen consumption (${\dot{\text{V}}}$O_2max_) test on a cycle ergometer (Velotron, SRAM, Chicago) was performed. Heart rate (HR) was monitored continuously during the ${\dot{\text{V}}}$O_2max_ test. A Cosmed (Quark CPET, Rome) was used to measure respiratory gas exchange (breath-by-breath) and was calibrated before each test. The test comprised a 5-min warm-up at 100 W followed by step increases of 50 W every 2.5 min. Once HR reached 165 BPM, or if respiratory exchange ratio reached 1.00, steps of 25 W per 2.5 min were used until voluntary exhaustion. ${\dot{\text{V}}}$O_2max_ data were then averaged in 10-s epochs for analysis to determine ${\dot{\text{V}}}$O_2max._

Those meeting the pre-determined inclusion criteria (see below) attended another session in which fluid loss was estimated during 1 h of cycling to determine fluid intake for experimental trials. This was conducted under the same conditions as experimental trials, cycling at 55% ${\dot{\text{V}}}$O_2max_ in an environmentally controlled chamber at 34 °C, 65% RH, with a front-facing air velocity of ~ 4.5 m s^−1^, large-diameter, industrial fan, continuously on, at the same setting (Imasu IFS65M (650 mm), Hong Kong).

### Participants

Inclusion criteria comprised regularly exercising, male, no cardiovascular or kidney disease, no medications or supplements excepting vitamins or sports drinks, passed PAR-Q, and a ${\dot{\text{V}}}$O_2max_ ≥ 50 mL kg^−1^ min^−1^.

Eighteen participants responded to advertisement, 18 came to the lab for an initial visit, 16 passed the screening and ${\dot{\text{V}}}$O_2max_ criteria and began the study.

Based upon earlier work (Vrijens and Rehrer 1999) and the rate of plasma sodium change observed, with a similar protocol with ingestion of water versus a typical sports drink with 18 mmol sodium L^−1^, we calculated a sample size of 12 to detect a difference of 1.6 mmol L^−1^ h^−1^, with standard deviation of 1.5 mmol L^−1^ h^−1^, α = 0.05, power = 0.90, for paired samples, and a sample size of 10 for power = 0.80.

### Experimental trials

Each participant consumed a diet of his choice before the first trial but was required to eat this same diet before the second trial. The last meal was consumed 2 h before each trial. Participants were also instructed to drink 1 L of a commercial sports drink (6 g L^−1^ carbohydrate, 21 mmol L^−1^ sodium and 13 mmol L^−1^ potassium) the evening before each trial and refrain from exercise for 24 h, and competition for 7 d, prior to each trial. On the day of the trial, they could drink water ad libitum prior to coming to the laboratory.

The trials were planned to consist of cycling for three hours at 55% ${\dot{\text{V}}}$O_2max_, during which they consumed either a chilled sports drink (Gatorade^®^ Lemon Lime) with sodium concentration of 21 mmol L^−1^ (Low Na^+^) or the same sports drink with added NaCl to achieve a sodium concentration of 60 mmol L^−1^ (High Na^+^). At least seven days separated trials, which were conducted at the same time of day for each participant.

Participants arrived approximately ~ 1 h before exercise was to start. They first voided their bladders and collected a urine sample, for urine specific gravity (USG) measurement to assess hydration status, and then were weighed. Thereafter, a rectal thermistor was inserted (11 cm past the anal sphincter), cycling clothes were put on and a heart rate monitor was attached. A venous catheter was placed in an antecubital vein and, after sitting for 10 min, the first baseline blood sample and other baseline physiological and subjective measures were taken. Participants then moved into the climate-controlled chamber and began cycling.

The beverage was consumed every 15 min (from 15–165 min) at a rate equal to the fluid loss, determined in the pre-trial.

Core temperature and heart rate (Polar Electro, Finland) were monitored continuously and recorded at 15 min intervals along with ratings of perceived exertion (RPE) (Borg scale 6–20) (3), body temperature sensation (scale 1–13) and thirst (scale 1–9). Subjective ratings were collected prior to beverage consumption and any blood draw.

Nude body mass (after voiding and drying) was also recorded before and after any trips to the toilet and at the end of each trial. Urine was collected, volume and USG of the post-exercise micturition were measured. Some urine samples were not collected. Criteria for terminating a trial before the planned three hours were: (1) a plasma sodium ≤ 130 mmol L^−1^; (2) core (rectal) temperature > 39.5 °C; heart rate reaching age-predicted maximum or (3) volitional exhaustion.

### Blood sampling and analyses

During each trial, venous blood samples were obtained from an indwelling catheter (22G, Becton Dickenson, Madrid, Spain) in an antecubital vein. Blood was drawn every 30 min during, and upon completion of, exercise. In total, during each experiment, ~ 30 ml of blood was drawn. Blood samples were analyzed immediately for sodium (GEM Premier 3000, Instrumentation Laboratory, Bedford, USA) and hematocrit and hemoglobin (OSM 3, Radiometer Medical, Brønshøj, DK). Changes in plasma volume (PV) were calculated according to Dill and Costill (Dill and Costill 1974).

Sweat loss was estimated by change in body mass corrected for mass loss from toileting and fluid intake. Rates of sweat loss were calculated by dividing by exercise time.

### Data analyses

Data are expressed as mean ± standard deviation. A *p* < 0.05 was accepted as statistically significant. *T*-tests were performed to analyze differences between conditions in fluid intake, change from baseline to end of exercise in body mass, plasma sodium concentration, plasma volume, estimated sweat loss, USG and exercise time. A linear fixed effects mixed model was used to analyze for any effects of condition, time and order for variables measured across the trial. SPSS (v26, IBM, Chicago) was used for these statistical analyses. Effect Size was calculated as a Cohen’s *d* (Mean Diff/ Average SD). Effect size (Cohen’s *d*) of 0.2 was considered small, 0.5 moderate and 0.8 large. Confidence intervals were calculated for differences between treatments in change (baseline − end) for primary outcome variables (plasma volume, plasma sodium concentration).

Dataset analyzed in the current study is available from the corresponding author upon reasonable request.

## Results

### Participants, trial completion and compliance

Three participants dropped out after the 1 h pre-trial because of time commitments. Another participant dropped out, because he fainted in the first trial after half an hour. He came back to repeat the trial, but felt unwell again and exited the study. Twelve participants conducted exercise trials in both conditions. One participant did not adhere to the treatment protocol having drunk significantly less than prescribed (% body mass loss > 2 × SD) and his data were, therefore, removed from all analyses. The 11 males who were included in results had a mean age of 36 ± 14 y, mass of 75.36 ± 5.30 kg, height of 180 ± 6 cm and ${\dot{\text{V}}}$O_2max_ of 60 ± 3 mL min^−1^ kg^−1^. Mean workload at 55% ${\dot{\text{V}}}$O_2max_ was 160 ± 23 W. Three participants did not complete 3 h exercise on High Na^+^ and two on Low Na^+^. Reasons for not completing the trials were exceeding 39.5 °C core temperature (*n* = 2), volitional exhaustion (*n* = 2), and reaching the maximal age-predicted HR (*n* = 1). Mean exercise time was similar in both experimental trials (176 ± 9 min Low Na^+^; 176 ± 7 min High Na^+^).

### Fluid balance

Participants (*n* = 10 Low Na^+^, *n* = 11 High Na^+^) were euhydrated prior to testing, as indicated by urine specific gravity (USG) and was similar (*p* = 0.53) in both conditions (1.016 ± 0.010 Low Na^+^; 1.014 ± 0.007 High Na^+^). USG at the end of exercise (*n* = 8 Low Na^+^, *n* = 10 High Na^+^) was (1.018 ± 0.008 Low Na^+^; 1.017 ± 0.004 High Na^+^) with no difference between conditions (*p* = 0.84, *d* = 0.10).

Similar fluid intakes, as well as estimated sweat losses, were observed between conditions (Table 1). Body mass loss was not significantly different between conditions, although there was a trend for the net loss to be less on High Na^+^, with a moderate effect size (Table 1).

**Table 1:** Plasma volume changes, fluid intakes, fluid losses and body mass changes (mean ± SD) in males (*n* = 11) during cycling^a^ in the heat with high (60 mml L^−1^) and low (21 mmol L^−1^) beverage sodium concentration

	Low Na^+^	High Na^+^	*p*^c^	Cohen’s *d*
Plasma volume change (%)	− 2 ± 2	0 ± 3	0.01	0.80
Rate of plasma volume change (% h^−1^)	− 0.6 ± 0.6	0.1 ± 1.1	0.01	0.76
Total fluid intake (L)	3.296 ± 0.582	3.353 ± 0.666	0.45	0.09
Rate of fluid intake (L h^−1^)	1.12 ± 0.19	1.14 ± 0.21	0.17	0.09
Estimated sweat loss^b^ (body mass loss + fluid in − toilet loss) (L)	3.28 ± 0.70	3.31 ± 0.49	0.84	0.05
Rate of estimated sweat loss^b^ (body mass loss + fluid in − toilet loss) (L h^−1^)	1.12 ± 0.23	1.13 ± 0.15	0.82	0.06
Body mass change (kg)	− 0.40 ± 0.30	− 0.22 ± 0.35	0.09	0.54
Body mass change (%)	− 0.53 ± 0.40	− 0.30 ± 0.45	0.09	0.55
Rate of body mass change (kg h^−1^)	− 0.137 ± 0.103	− 0.078 ± 0.117	0.10	0.55

^a^3 h or until exhaustion

^b^*n* = 7 Low Na + , *n* = 8 High Na ^+^

^c^Paired *T*-test except for sweat loss, (unpaired), as four final urine volume measures were omitted in Low Na + and three in High Na ^+^

### Plasma sodium

**Fig. 1:**
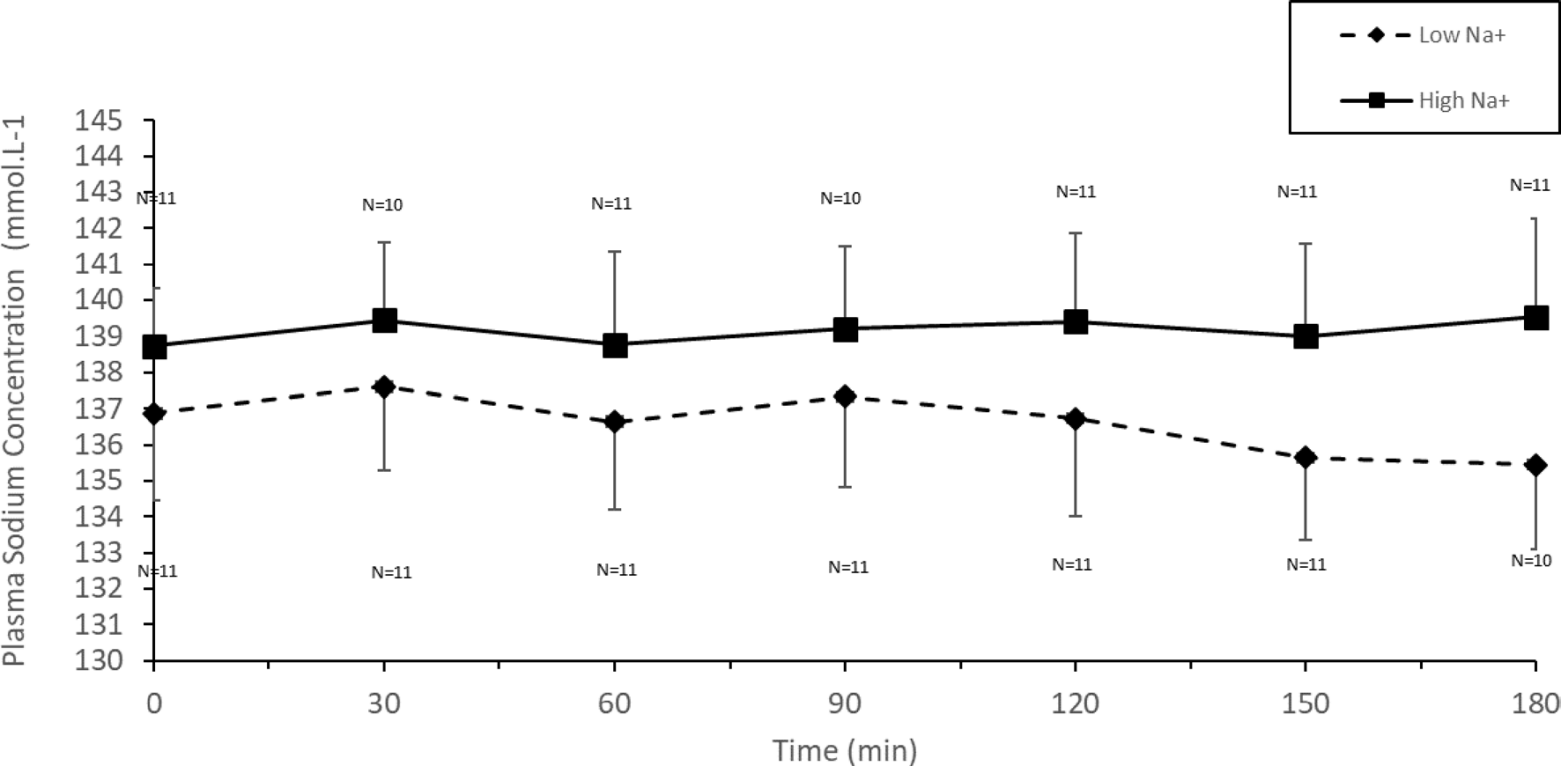
Plasma sodium concentration in males during cycling (55% ${\dot{\text{V}}}$O_2max_) in the heat (34 °C, 65% RH) in which fluid replacement was with either a sports drink with 21 mmol L^−1^ (Low Na^+^) or the same sports drink with 60 mmol L^−1^ (High Na^+^)

Plasma sodium concentration over the course of exercise trials was greater with High Na^+^ than Low Na^+^ (Fig. 1) (*p* < 0.001). Change in plasma sodium from baseline to final measure was −1.5 ± 2.2 mmol L^−1^ on Low Na^+^ and 0.8 ± 2.4 mmol L^−1^ on High Na^+^ (*p* = 0.048, 95% CI [−4.52, −0.02], *d* = 0.99) (Individual data can be found in Supplementary Fig. 6.) Four participants had plasma sodium < 135 on Low Na^+^ and one on High Na^+^.

### Plasma volume change

**Fig. 2:**
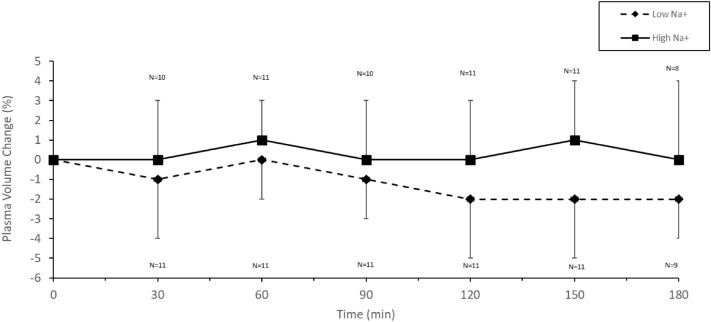
Change in plasma volume in males during cycling (55% ${\dot{\text{V}}}$O_2max_) in the heat (34 °C, 65% RH) in which fluid replacement was with either a sports drink with 21 mmol L^−1^ (Low Na^+^) or the same sports drink with 60 mmol L^−1^ (High Na^+^)

Plasma volume decreased over the course of exercise with Low Na^+^ but not with High Na^+^ (Fig. 2) (*p* < 0.001). Change in plasma volume from baseline to final measure was also significantly different with Low Na^+^ versus High Na^+^ (*p* = 0.02, 95% CI [−3.2, −0.5]) with a large effect size (Table 1) (Individual data can be found in Supplementary Fig. 7.)

### Heart rate, thermal and subjective responses

**Fig. 3:**
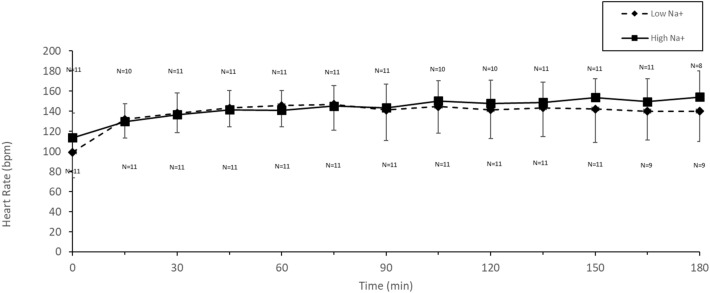
Heart rate in males during cycling (55% ${\dot{\text{V}}}$O_2max_) in the heat (34 °C, 65% RH) in which fluid replacement was with either a sports drink with 21 mmol L^−1^ (Low Na^+^) or the same sports drink with 60 mmol L^−1^ (High Na^+^)

Heart rate increased over time in both trials (Fig. 3; *p* < 0.0001) with no significant difference between conditions (*p* = 0.11) nor interaction (*p* = 0.95).

**Fig. 4:**
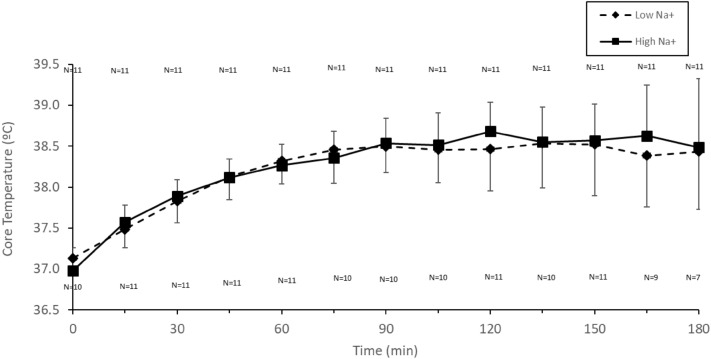
Core temperature (°C) in males during cycling (55% ${\dot{\text{V}}}$O_2max_) in the heat (34 °C, 65% RH) in which fluid replacement was with either a sports drink with 21 mmol L^−1^ (Low Na^+^) or the same sports drink with 60 mmol L^−1^ (High Na^+^)

Mean rectal temperature increased over time (*p* < 0.001), with no significant condition effect (*p* = 0.37) (Fig. 4) nor interaction (*p* = 0.94), but there was an order effect with temperature being greater in the second trial (*p* < 0.001).

**Fig. 5:**
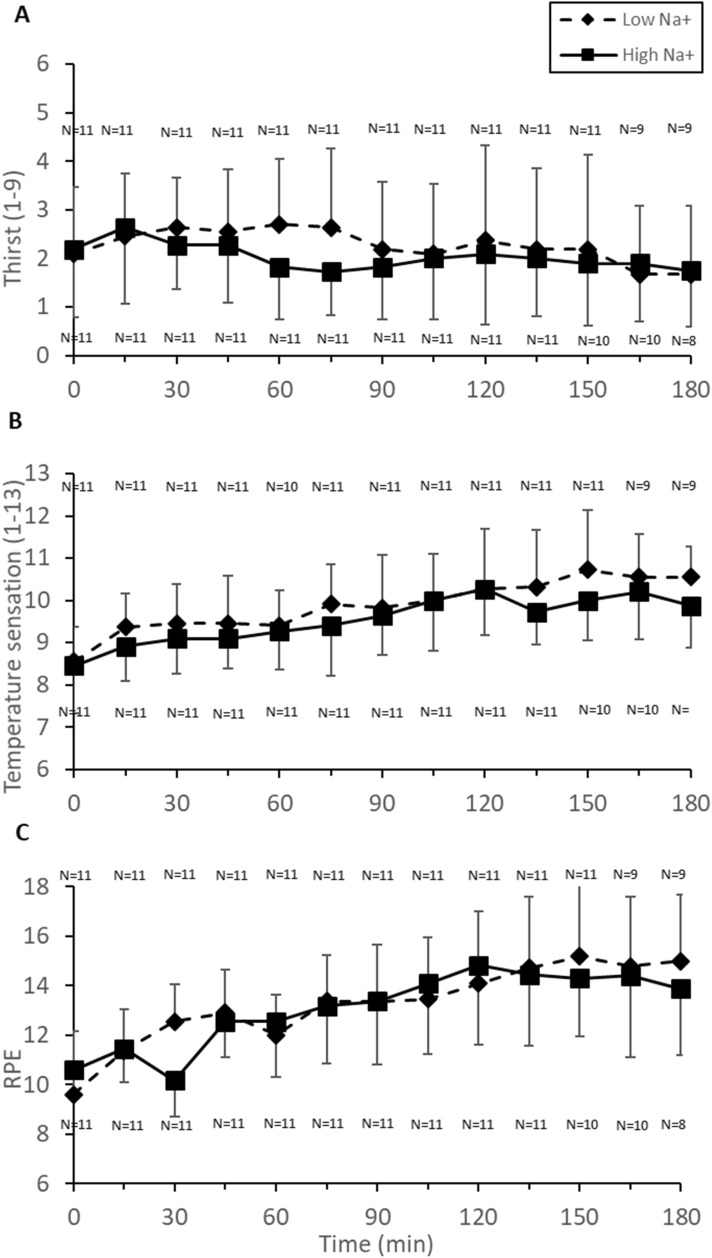
Subjective measures of thirst, warmth and perceived exertion in males during cycling (55% ${\dot{\text{V}}}$O_2max_) in the heat (34 °C, 65% RH) in which fluid replacement was with either a sports drink with 21 mmol L^−1^ (Low Na^+^) or the same sports drink with 60 mmol L^−1^ (High Na^+^)

Thirst was not different between conditions (*p* = 0.14) nor did it change over time (*p* = 0.64) (Fig. 5), however, there was an order effect with thirst being greater in the first trial (p < 0.001).

Participants felt hotter in Low Na^+^ than High Na^+^ (*p* = 0.002), over time (*p* < 0.001), and in the second trial (*p* < 0.001) (Fig. 5), but no interaction was evident (*p* = 0.97). Ratings of perceived exertion increased over time (*p* < 0.001) with no difference between conditions (*p* = 0.71) (Fig. 5) nor significant interaction (*p* = 0.94).

## Discussion

The current finding of maintaining plasma sodium concentration and plasma volume when fluid loss was replaced with a beverage having a sodium concentration of 60 mmol L^−1^ versus one with 21 mmol L^−1^, in which decreases were observed, supports the body of knowledge indicating that sodium has a clear role in the maintenance and reinstatement of fluid and sodium balance with long lasting, moderate intensity exercise in the heat (Sanders et al. 2001; Shirreffs et al. 1996). Our study demonstrates that plasma sodium can be maintained during exercise, when fluid intakes nearly match sweat losses, if acute sodium concentration is increased to the mid-upper range of sweat sodium concentrations observed with high sweat rates (Hamouti et al. 2011; Baker et al. 2009). Whether this approach is applicable to the prevention of exercise associated hyponatremia is controversial. Overconsumption of (hypotonic) fluids is the primary contributing factor in exercise associated hyponatremia (Hew-Butler et al. 2017) even in the absence of fluid gain (Chlíbková et al. 2016), however, the role of sodium is contested (Hew-Butler et al. 2017). By not replacing sweat losses accrued during exercise, or only partially replacing them with hypotonic fluids, plasma sodium concentration will typically rise, despite a net sodium loss, the extent of which is dependent upon the volume and sodium concentration of the sweat loss and that of any ingested fluids, and renal water and sodium retention.

Sodium has direct and indirect effects to regulate plasma volume which can affect exercise performance and strain, particularly due to effects on stroke volume and cardiac output (Luetkemeier and Thomas 1994) and concomitant effects on heart rate (Montain and Coyle 1992b). In warm environments, a decreased plasma volume can also impact thermoregulation (Sawka et al. 2001) with increased osmolarity acting independently of plasma volume to decrease skin blood flow and increase thermal (Montain and Coyle 1992a) and cardiovascular strain (Chou et al. 2018). In the present study, plasma volume was maintained with the High Na^+^ beverage, and reduced with the Low Na^+^ beverage, this despite the fact that a similar volume of fluid was ingested. The maintenance of plasma volume with High Na^+^ reflects the effect that sodium has on fluid retention and extracellular fluid volume. Similar findings were observed by Sanders et al. (2001) who found, under milder conditions (20 °C) but similar protocol, that ECF was better maintained with ingestion of a beverage having a sodium concentration of 50 mmol L^−1^, decreased with 5 mmol L^−1^ and expanded with 100 mmol L^−1^. Associated with this was a reduction in free water clearance, with both 50 and 100 mmol L^−1^, more so with the latter.

In line with our plasma volume results, body mass loss also tended to be less with High Na^+^, with a moderate effect size. Although there was no difference in heart rate between conditions, if similar fluid losses and replacement were continued over a longer period a possible further decrease in plasma volume with Low Na^+^, due to a greater net sodium loss, and associated increase in heart rate, might be anticipated. Although differences observed were not of a magnitude to have clinical significance with health impacts, effects on performance, particularly if exercise was longer or more intensive, might be expected.

With shorter exercise endeavors than in the present study, lower intensity and/ or cooler temperatures, sweat rate, sweat sodium concentration and total sweat loss would be less (Baker 2017). In such situations, or in types of events in which other foods with adequate sodium are ingested, an effect of different beverage sodium content on plasma sodium or plasma volume would not be expected.

The lack of difference in heart rate and core temperature between conditions in the present study correlates with the similar RPE observed, however, unexpectedly, perceived warmth was greater with Low Na^+^. Thirst was also similar in the two conditions, although it was lower in the second trial despite no differences in fluid intakes or net balance, possibly indicating a psychological component of conditioning with repeated exposure. The lack of a physiological explanation for the reduced thirst in the second trial underscores the fact that thirst is not always simply related to osmolarity and volume/blood pressure changes. These signals are interfaced with conscious perceptions in a complex dynamic manner (Armstrong et al. 2020).

Advocating “drinking to thirst”, while thirst is not always associated with a given level of hydration (Armstrong et al. 2020), is contentious. There does not, however, appear to be an exact cut-off in terms of fluid (% body mass) loss that coincides with performance decrease, and there is even less consensus whether drinking to thirst impairs performance or not, compared to prescribed drinking (Bardis et al. 2013; Hoffman and Stuempfle 2016; Kenefick 2018). It is, however, known that exercise can blunt thirst (Greenleaf 1992), that sweat rate can be substantially greater than the rate of gastric emptying (Mitchell and Voss 1991; Torii 1995) and that effects of hypohydration and the associated reduction in extracellular fluid and plasma volume can decrease performance, particularly in the heat (Coyle 2004).

Although not replacing sweat losses and allowing hypohydration to ensue is one way to ensure that plasma sodium concentration is not reduced, this may be at the expense of total body and extracellular water, and plasma volume. For the recreational athlete this may be acceptable, when maintaining a given intensity or performance is not critical. This may even be preferable to risking hyponatremia in the large group of non-competitive endurance event participants that are most at risk. For competitive endurance athletes, particularly in the heat, sweat rates can exceed 2 L h^−1^ (Convertino et al. 2007; Rehrer and Burke 1996). With maximal gastric emptying rates being around 1.2 L h^−1^ (Mitchell and Voss 1991), drinking more than this is superfluous, and may cause gastrointestinal discomfort (Mitchell and Voss 1991). In this situation sodium intake sufficient to replace losses is key to retaining fluid, decreasing urinary losses, and thereby enhancing reinstatement of extracellular fluid volume.

### Limitations

Our findings are based on a situation in which large fluid losses are replaced with large fluid intakes and would not necessarily be applicable to those who do not lose and replace a considerable amount of body fluid during exercise, or in those who ingest considerable amounts of sodium in other foods or supplements in conjunction with fluid replacement. We also do not know if similar responses would be found in those heat acclimatized.

## Conclusions

In endurance events lasting ~ 3 h (or more), athletes who experience significant sweat losses, and compensate them with fluid intake, may benefit from increasing the sodium concentration of beverages consumed. Beverages with increased sodium (60 mmol h^−1^) can maintain plasma sodium concentration and plasma volume whereas they both decrease when replacing sweat losses with beverages having a sodium profile typically found in sports drinks.

## Supplementary Information

Below is the link to the electronic supplementary material.

Supplementary file1 (DOCX 13 KB)

Supplementary file2 (TIFF 80 KB)

Supplementary file3 (TIFF 28 KB)

## Abbreviations

PAR-Q: Physical activity readiness questionnaire
${\dot{\text{V}}}$O_2max_: Rate of maximal oxygen consumption
HR: Heart rate
BPM: Beats per minute
RPE: Rating of perceived exertion
USG: Urine specific gravity
RH: Relative humidity
PV: Plasma volume
SD: Standard deviation

## References

[CR1] Anastasiou CA, Kavouras SA, Arnaoutis G, Gioxari A, Kollia M, Botoula E, Sidossis LS (2009). Sodium replacement and plasma sodium drop during exercise in the heat when fluid intake matches fluid loss. J Athl Train.

[CR2] Armstrong LE, Giersch GE, Dunn L, Fiol A, Muñoz CX, Lee EC (2020). Inputs to thirst and drinking during water restriction and rehydration. Nutrients.

[CR3] Baker LB (2017). Sweating rate and sweat sodium concentration in athletes: a review of methodology and intra/interindividual variability. Sports Med.

[CR4] Baker LB, Stofan JR, Hamilton AA, Horswill CA (2009). Comparison of regional patch collection vs. whole body washdown for measuring sweat sodium and potassium loss during exercise. J Appl Physiol.

[CR5] Bardis CN, Kavouras SA, Arnaoutis G, Panagiotakos DB, Sidossis LS (2013). Mild dehydration and cycling performance during 5-kilometer hill climbing. J Athl Train.

[CR6] Burke LM, Hawley JA, Wong SHS, Jeukendrup AE (2011). Carbohydrates for training and competition. J Sports Sci.

[CR7] Chatterjee A, Abraham J, Grumezescu AM, Holban AM (2019). 15—a comprehensive study on sports and energy drinks. Sports and energy drinks.

[CR8] Chlíbková D, Rosemann T, Posch L, Matoušek R, Knechtle B (2016). Pre-and post-race hydration status in hyponatremic and non-hyponatremic ultra-endurance athletes. Chin J Physiol.

[CR9] Chou T-H, Allen JR, Hahn D, Leary BK, Coyle EF (2018). Cardiovascular responses to exercise when increasing skin temperature with narrowing of the core-to-skin temperature gradient. J Appl Physiol.

[CR10] Convertino VA, Armstrong LE, Coyle EF, Mack GW, Sawka MN, Senay LCJ, Sherman WM (2007). Exercise and fluid replacement. Med Sci Sports Exerc.

[CR11] Coombes JS, Hamilton KL (2000). The effectiveness of commercially available sports drinks. Sports Med.

[CR12] Coyle EF (2004). Fluid and fuel intake during exercise. J Sports Sci.

[CR13] Dill DB, Costill DL (1974). Calculation of percentage changes in volumes of blood, plasma, and red cells in dehydration. J Appl Physiol.

[CR14] Greenleaf JE (1992). Problem: thirst, drinking behavior, and involuntary dehydration. Med Sci Sports Exerc.

[CR15] Hamouti N, Del Coso J, Ortega JF, Mora-Rodriguez R (2011). Sweat sodium concentration during exercise in the heat in aerobically trained and untrained humans. Eur J Appl Physiol.

[CR16] Hew-Butler T, Loi V, Pani A, Rosner MH (2017) Exercise-associated hyponatremia: 2017 update. Front Med 4 (21). 10.3389/fmed.2017.00021 10.3389/fmed.2017.00021 PMC5334560 28316971

[CR17] Hoffman MD, Stuempfle KJ (2016). Is sodium supplementation necessary to avoid dehydration during prolonged exercise in the heat?. J Strength Cond Res.

[CR18] Kenefick RW (2018). Drinking strategies: planned drinking versus drinking to thirst. Sports Med.

[CR19] Koenders E, Franken C, Cotter J, Thornton S, Rehrer N (2017). Restricting dietary sodium reduces plasma sodium response to exercise in the heat. Scand J Med Sci Sports.

[CR20] Luetkemeier MJ, Thomas EL (1994). Hypervolemia and cycling time trial performance. Med Sci Sports Exerc.

[CR21] Maughan RJ, Leiper JB (1995). Sodium intake and post-exercise rehydration in man. Eur J Appl Physiol.

[CR22] Merson SJ, Maughan RJ, Shirreffs SM (2008). Rehydration with drinks differing in sodium concentration and recovery from moderate exercise-induced hypohydration in man. Eur J Appl Physiol.

[CR23] Mitchell JB, Voss KW (1991). The influence of volume on gastric emptying and fluid balance during prolonged exercise. Med Sci Sports Exerc.

[CR24] Montain SJ, Coyle EF (1992). Fluid ingestion during exercise increases skin blood flow independent of increases in blood volume. J Appl Physiol.

[CR25] Montain SJ, Coyle EF (1992). Influence of graded dehydration on hyperthermia and cardiovascular drift during exercise. J Appl Physiol.

[CR26] Nadel ER, Fortney SM, Wenger CB (1980). Effect of hydration state of circulatory and thermal regulations. J Appl Physiol.

[CR27] Rehrer NJ, Burke LM (1996). Sweat losses during various sports. Aus J Nutr Diet.

[CR28] Rehrer N, Wagenmakers A, Beckers E, Halliday D, Leiper J, Brouns F, Maughan R, Westerterp K, Saris W (1992). Gastric emptying, absorption, and carbohydrate oxidation during prolonged exercise. J Appl Physiol.

[CR29] Sanders B, Noakes TD, Dennis SC (2001). Sodium replacement and fluid shifts during prolonged exercise in humans. Eur J Appl Physiol.

[CR30] Sawka MN, Montain SJ, Latzka WA (2001). Hydration effects on thermoregulation and performance in the heat. Comp Biochem Physiol A Mol Integr Physiol.

[CR31] Shirreffs SM, Taylor AJ, Leiper JB, Maughan RJ (1996). Post-exercise rehydration in man: effects of volume consumed and drink sodium content. Med Sci Sports Exerc.

[CR32] Sims ST, van Vliet L, Cotter JD, Rehrer NJ (2007). Sodium loading aids fluid balance and reduces physiological strain of trained men exercising in the heat. Med Sci Sports Exerc.

[CR33] Strydom N, Holdsworth L (1968). The effects of different levels of water deficit on physiological responses during heat stress. Int Zeitschr Angew Physiol Einsch Arbeitsphysiol.

[CR34] Torii M (1995). Maximal sweating rate in humans. J Hum Ergol.

[CR35] Vrijens DMJ, Rehrer NJ (1999). Sodium-free fluid ingestion decreases plasma sodium during exercise in the heat. J Appl Physiol.

