# Supplementary material for: A randomized, cross-over trial assessing effects of beverage sodium concentration on plasma sodium concentration and plasma volume during prolonged exercise in the heat
Source: Eur J Appl Physiol. 2022 Sep 29;123(1):81–9. doi: 10.1007/s00421-022-05025-y (PMC9813217; doi:10.1007/s00421-022-05025-y)
Supplement: Supplementary file 1 — Supplementary file1 (DOCX 13 KB) [file 421_2022_5025_MOESM1_ESM.docx]

Legends to supplementary figures

Figure 6. Individual changes in plasma sodium concentration from baseline to exercise completion, in males (n=11), during cycling (55 % V̇O_2max_) in the heat (34° C, 65% RH). in which fluid replacement was with a sports drink with 21 mmol sodium. L^-1^ (Low Na^+^) or the same sports drink with 60 mmol sodium. L^-1^ (High Na^+^).

Figure 7. Individual changes in plasma volume from baseline to exercise completion, in males (n=11), during cycling (55 % V̇O_2max_) in the heat (34° C, 65% RH). in which fluid replacement was with a sports drink with 21 mmol sodium. L^-1^ (Low Na^+^) or the same sports drink with 60 mmol sodium. L^-1^ (High Na^+^).
