# Supplementary figures and images for: A randomized, cross-over trial assessing effects of beverage sodium concentration on plasma sodium concentration and plasma volume during prolonged exercise in the heat
Source: Eur J Appl Physiol. 2022 Sep 29;123(1):81–9. doi: 10.1007/s00421-022-05025-y (PMC9813217; doi:10.1007/s00421-022-05025-y)

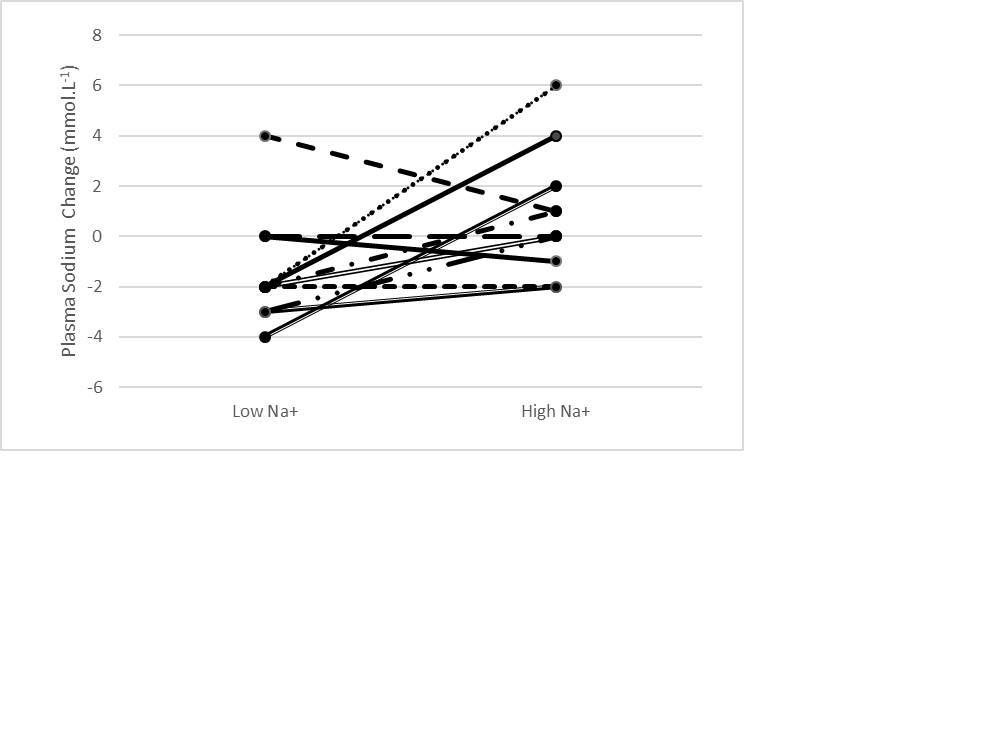

Supplement: Supplementary file 2 — Supplementary file2 (TIFF 80 KB) [file 421_2022_5025_MOESM2_ESM.tiff]
